# Maps and atlases of cancer mortality: a review of a useful tool to trigger new questions

**DOI:** 10.3332/ecancer.2016.670

**Published:** 2016-09-01

**Authors:** Alberto d’Onofrio, Chiara Mazzetta, Chris Robertson, Michel Smans, Peter Boyle, Mathieu Boniol

**Affiliations:** 1 International Prevention Research Institute, Lyon 69006, France; 2 IstitutoEuropeo di Oncologia Milano 20141, Italy; 3 Strathclyde University, Glasgow G1 1XQ, Scotland, UK; * Chiara passed away in November 2010

**Keywords:** cancer, atlas, epidemiology, geography, spatial statistics, tobacco-related cancers, alcohol-related cancers, breast cancer, geographic medicine

## Abstract

In this review we illustrate our view on the epidemiological relevance of geographically mapping cancer mortality.

In the first part of this work, after delineating the history of cancer mapping with a view on interpretation of Cancer Mortality Atlases, we briefly illustrate the ‘art’ of cancer mapping. Later we summarise in a non-mathematical way basic methods of spatial statistics.

In the second part of this paper, we employ the ‘Atlas of Cancer Mortality in the European Union and the European Economic Area 1993–1997’ in order to illustrate spatial aspects of cancer mortality in Europe. In particular, we focus on the cancer related to tobacco and alcohol epidemics and on breast cancer which is of particular interest in cancer mapping.

Here we suggest and reiterate two key concepts. The first is that a cancer atlas is not only a visual tool, but it also contain appropriate spatial statistical analyses that quantify the qualitative visual impressions to the readers even though at times revealing fallacy. The second is that a cancer atlas is by no means a book where answers to questions can be found. On the contrary, it ought to be considered as a tool to trigger new questions.

## Introduction

Epidemiology can be defined as the study of the distribution and of determinants of health-related states and events. As such mapping is intrinsic to epidemiology, and it is the first step for defining an epidemic. Not surprisingly, the relevance of geographical features to better understand a disease was first stressed by Hippocrates himself who also devoted a treatise to environmental determinants of diseases. The title of it was ‘Airs, Waters, and *Places*’ (italic is ours) [weblink: http://classics.mit.edu/Hippocrates/airwatpl.html].

Waters and places lie at the core of the birth of modern disease mapping was well elucidated in the celebrated work of J Snow during the London cholera epidemics in 1854. By mapping the cholera cases he was able to identify as the source of risk the water provided by the ‘Southwark and Vauxhall Water Company’, and in particular a hand pump located in Broad Street. The removal of the hand pump caused the end of the epidemics.

As far as cancer is concerned, a fundamental cornerstone of cancer epidemiology is ‘Cancer in five Continents’ by [19]. This publication clearly showed the geographic issues of cancer distribution and determinants, but it was not a cancer atlas.

Indeed, a cancer atlas can be defined as the geographical representationof cancer mortality and/or incidence on thematic maps that visually describe the cancer scenery of a country [21] or of a group of countries [10]. We remind the readers here that a thematic map focuses on displaying the geographic occurrence and variation of a single phenomenon, and hence is called **the theme** of the map.

To the best of our knowledge, the first cancer atlas was Haviland’s maps of cancers in women in England and Wales, published in 1875 [25]. It started as an idea, possibly quite a deterministic one, that ‘by studying the geographical laws of disease we shall know where to find its exciting as well as its predisposing causes and how to avoid it’. It was fixed on the observation derived from ‘definite character of the arrangement that the mortality assumes throughout the country’. Haviland tried to associate the cancer distribution to the distribution of some mineral contents in the areas. Stocks in the 1930s also performed similar investigation [54–57]. Howe in 1963 published a national cancer atlas for UK [26].

A landmark for modern geographical epidemiology of cancer was the cancer atlas based on the then 49 states of the USA by Burbank [12]. Firstly, it was the first computer-drawn atlas of cancer. The use of computers has obviously given a huge impetus to the researches in this area. Secondly, it was important to understand that the average size of a US state is too large a spatial unit and that smaller areas (e.g. counties in the USA) are more appropriate for epidemiological investigation. This led in to the publication of USA cancer atlases at the level of USA counties [35–36]. A remarkable history and critical review of the 1975 USA and following cancer atlases has been published by Pickle [49] which is an excellent starting point for deepening this important topic.

Another pioneering important cancer atlas has been the atlas of cancer mortality in the People’s Republic of China [20, 33], which reported data taken from the period 1973–1975.

The USA cancer atlases by Mason and co-workers are also important because they led to identification of some previously unknown risk factors for some cancers [62]. It is interesting to mention the case of oral cancers in women because it sheds light on the fact how cancer mapping must be considered as a trigger for questions whose response needs a careful investigation, i.e. a need for complex research process that must go beyond first impressions. The USA atlases helped in the identification of areas in the southwest of the USA that had cluster of large mortality rates for oral cancers in women. These data had a good geographical correlation with the presence of the textile industry, where the majority of workers were women. This initially led to the formulation of the hypothesis of the presence of some work-related exposures, i.e. a professional risk. More detailed investigations showed that in these industries cigarette smoking was prohibited, and hence as an alternative to tobacco smoking, the workers use to chew tobacco.

This and other findings stimulated by the US 1975–1976 atlases led to a significant Public Health ‘action to reduce cancer rates and health disparities’ [46].

Another outstanding example comes from the Chinese atlas [20, 33] which allowed the identification of the Linxian region, where there was a cluster of very large prevalence and mortality for oesophageal and gastric cancers. This concurred with the identification of lack of important micronutrient in the diet among the risk factors [58].

As far as risk factors are concerned, it is interesting to mention a possible ‘reverse approach’ based on cancer mapping: the map of lung cancer in Switzerland has been recently used to indirectly identify spatial patterns, and the main risk factor found being smoking [29].

Many national cancer atlases followed the pioneering one by Burbanks in the immediately following years. For example, as early as in 1992, Smans and colleagues listed at least 28 national atlases [62]. Among them, noteworthy are the ‘second generation’ US ancer Atlas edited in 1987 and 1990 [44–45], which followed the methodologies proposed by Mason and coworkers [36–37]. Moreover, in 1989 the first book had been published summarising the state of the art of practical and theoretical issues related to cancer mapping [8].

Since then a very large number of National Cancer Atlases for various countries and very large regions within states have been published, among which are: USA [18], Spain [35] Brazil [39], Colombia [42], Queensland state of Australia [14–15], Saudi Arabia [3], Argenitina [38, 1].

However, important national atlases of cancers have a relevant intrinsic limitation. Indeed, cancer determinants and their related spatial patterns can span across multiple nations. They are not constrained, in general, by national borders. An important recent example is the mapping and investigation of gastric cancer incidence and mortality in Latin America [59], which showed as incidence and mortality of this very common cancer span across multiple nations, following patterns that are well correlated with the mountain patterns. It was shown, thus, that altitude is associated with the burden of gastric cancer. In turn, the authors suggest that altitude is a good proxy of other risk factors such as environmental, genetic, and bacterial (*H. pylori*).

The necessity and importance of producing multinational cancer atlases is therefore demonstrated. One of the first multinational cancer atlas has been the World Health Organisation (WHO) Atlas of Cancer Mortality in the nine countries of the then European Economic Community [62], which was published in 1992 and referred to the period between late 70 and early 80s.

A spatially detailed atlas of Cancer incidence and mortality in northern European countries between 1981 and 1990 was published in 2001 [47].

In 2009, a new atlas for cancer mortality was published by WHO [10], where mortality data were mapped for the period 1993–1997 from all the 25 member states of the European Union (as of May 2004) and for three member states (Iceland, Norway, and Switzerland) of the European Economic Area (EEA). As far as the adopted geographical units are concerned, the 2009 atlas included 1278 areas designated as being at levels II or III as defined by the European Commission (EC) statistical services. Thus, the information contained in this second WHO European Atlas showed a marked quantitative and qualitative increase with respect to the one included in the above-mentioned previous Atlas [62].

The second WHO cancer atlas for Europe based on over 5.5 million deaths from cancer and 2.2 billion person-years of observation, revealed many distinctive patterns of cancer mortality distribution within the then European Union and the European Economic Area (EU–EEA).

Cancer atlases are, of course, of relevance because visual information is the most immediate and important kind of information. It is like saying among the ancient Greeks the past tense *oida* of the verb ‘to see’ (*orao*) meant also ‘to know’: *oida* = *I know* because *I have seen* [60]. However, visual information alone needs careful interpretation and validation. For this reason an atlas of cancer is not meant to be a tool providing answers. On the contrary, it is a tool suggesting questions, and here lies probably the main interest of a cancer atlas.

Indeed, cancer occurs in people and not in geographical areas [10]. Thus a cancer atlas is aimed for providing not only qualitative-visual and analytical identification of geographical areas that require more detailed study but also for the formulation of aetiological hypotheses (to be then validated, of course) in order to account for the observed differences. It should be noted that the identification is not only visual but also analytical. Indeed, an epidemiological atlas provides not only maps but also more or less detailed statistics concerning the spatial and if possible statistics on how spatial features of the disease in study changes during the time.

The impact of cancer atlases is not only limited to the field of the aetiology of diseases. Indeed, they can also be a useful practical tool for public health planning: the identification of areas of anomalous cancer occurrence/mortality can suggest a better spatial allocation of resources as well as localised targeted interventions aimed at cancer prevention. Again, also in the field of public health planning, a cancer atlas has the aim of triggering questions, in this specific case concerning the quality and effectiveness of current interventions.

Of course the above considerations on maps and on their meaning and aims apply not only to cancer mapping but to the geographical representation and investigation of other diseases. Indeed, recently a new discipline has been born at the interface between Medicine, Epidemiology, and Geography named Geographical Medicine [52, 23, 11].

This paper is organised as follows: first we introduce a number of issues that are at the base of cancer mapping, which we summarise it in the title ‘The ’Art’ of Cancer Mapping’, and then we review the main tools of statistical analysis of cancer maps. Later we also illustrate two paradigmatic examples of geographical epidemiological analyses. First we illustrate the geographic impact on cancer mortality of tobacco and alcohol, and then the geography of breast cancer mortality in Europe. A discussion on these findings concludes this work.

We shall use the above-mentioned 2009 Atlas of Cancer Mortality [10] as reference work to illustrate the main features of cancer mapping as well as patterns of geographical distribution of the mortality rates for a selected number of cancer in the time period 1993–1997. For this reason, here we use the term cancer atlas to denote Cancer Mortality Atlas, unless otherwise specified.

## The ‘art’ of cancer mapping

### Identifying and coding: the most subtle art

The first major issue in mapping cancer mortality is data collection and homogenisation. Indeed, cancer mortality data suffer from intrinsic problems related to death certification, which often do not correctly report the real cause of the death. Putting a label to a death can be a difficult task not only for the person filling in a death certificate but also for the final classification of the death. Namely, especially in the past, in the absence of clear rules the reported cause of death was not necessarily the primary cancer but the metastatic site or some important cancer-related diseases (e.g. pneumonia for lung cancer). Rules have evolved to consider as much as possible the underlying cause of death, but this does not prevent diverse appreciations in different regions of what is the underlying cause of death.

A second related issue is the cause of death coding, the International Classification of Disease (ICD) system, which evolves through time in its various and increasingly detailed editions which can potentially be asynchronously adopted by the various countries in their death certification systems. This can be an important issue if the time span to be covered is quite long. In the long term, gradual compliance of national death certification rules and practices following WHO’s recommendations for establishing and well-coding underlying causes of death should greatly improve this critical issue.

Misclassification and other problems may synergise as there being various important cancer sites within. For example, despite to the existence of various and quite different-forms of leukaemia coded in the various versions of ICD, death certificates frequently report only ‘leukaemia’, without specifying the involved cell type. Accurate recording of the precise site of cancer of the large bowel is often difficult, which is mirrored by the fact that ICD9 included a code (ICD-9 153) for cancers of ‘intestinal tract, part unspecified’. This coding in ICD10 corresponds to C17.9 and C18.9. Deaths caused by cancers of the cervix and the body of the uterus (ICD-9 180 and 182, respectively) often appeared as cancer of the uterus on death certificates in several countries [16, 48]. The need to exactly identify and separate these two kinds of cancers has been discussed for many years [34]. Even with efforts in harmonising code, these are diversely used. While in ICD10, C54 denotes corpus uteri, C53 cervix uteri, and C55 unspecified parts, the code C55 is still frequently used by some countries and cause potential incomplete reporting of cervical cancer and corpus uteri mortality [34]. Finally, as far as the various forms of non-Hodgkin’s lymphoma (ICD-9 200 and 202, corresponding to ICD10 C82-86, C96) are concerned, there are national differences in the nomenclature and classification of these forms of malignant disease.

## Choosing the right geographical unit

Coming to issues that are specific to geographical mapping, a crucial problem is choosing the right areal units. These have to be the smallest possible but not too small, in order to avoid two problems caused by excessive smallness of areal units, i.e. the excess of between-areas randomness and the presence of false clusters of hot/cold spots in the data for the period in study. However, it is not possible for a mapper to freely choose the optimal features of the unit areas because at nation level data are aggregated in administrative units, which of course goes beyond the control of the mapper. Since the average areal size administrative divisions are nation-specific this can generate dishomogeneity between the different countries of a map. For example, counties in Germany have an average area smaller than the ones in Spain. A possible solution to the abovementioned problem might be the aggregation of small counties in larger geographical entities for statistical purposes in order to suppress noise and other artefacts. However, this operation would be extremely arbitrary, and it might induce false patterns or remove existing geographical major differences in the resulting maps.

### Use the right ‘theme’

To make a thematic map, a clear quantitative definition of the theme is fundamental. If the theme is the mortality for cancer one may choose among three options. The first is to use the crude mortality, which is defined as the number of deaths because of a given cancer (or set of cancers) in a given population for a specific time period, expressed as rate of deaths per annum per 100,000 persons. However, statistics has been developed by the way of standardising the above crude rate with respect to a given reference population with known age structure [9] in order to display actual difference in cancer mortality risks rather that displaying difference in age distribution of population. In [10] the direct standardisation was used, in which it adopts as reference population the world standard population [64]. The direct standardisation with a well-defined and internationally adopted reference population allowed both comparisons with data published elsewhere, and it also made it possible in an atlas to compare the mortality maps of different kinds of cancers. Finally, the third option is the so-called indirect standardisation which consists to apply the global mortality rate computed in the whole area of the atlas to compute mortality expected in each area and then standardise the observed mortality by computing the ratio observed to the expected. This way of standardising, however, does not allow for comparisons. It has advantages, however, in the presence of areas of very scarce population in general and/or in particular age-strata.

## The ‘Art’ of colouring

In a thematic map, its colouring, which visually represents the quantitative changes of the theme, is fundamental. However, if as in an atlas one has to deal with a set of such maps, the mapper has to choose between the use of a same colouring scale for all the maps (i.e. an ‘absolute’ scale) or to use different scales (i.e. scales relative, in case of a cancer atlas, to cancer and sex). In the case of a mortality atlas, the first choice is not the best from the visual point of view since the range of variation of cancer mortality is highly wide, and hence the cancer maps would result being quite uniform and be of scarce readability. This defect apparently annihilates the advantage of the use of uniform scale. In [10] each main map adopts a cancer and sex specific colouring. This makes the visual comparison of pairs and groups of maps less easy, but allows more easily the visual detection of patterns and of areas of randomness. However, in [10] also maps with common colouring (MCC) were included. Indeed, it is also important to show this kind of maps because in the case where the span of mortality rate is small, the cancer and sex specific colouring may give the visual impression of a large span of the data. See section 7 for a specific example: the mapping of breast cancer mortality.

The choosing of the colours, the number of them, and the law that associates a colour to a value range of the theme are a long debated issue in literature of thematic maps [30]. For example, in our reference atlas [10] the following conventions were adopted: *i) Colours:* the higher rates in shades of orange/red, the median rates in yellow, and the lower rates in shades of green ([30] for a fuller explanation); *ii) Numbers:* seven colours (21 for the maps with cancer-independent colouring); *iii) the law of correspondence:* seven (21 for MCC) intervals of quantiles with cut-points variable for each specific kind of cancer and sex in order to improve the visual understanding of each map.

### Art and artefacts

Finally it is worth mentioning that an intrinsic limit of mapping is the heterogeneous distribution of population. This heterogeneity may imply a wrong perception of risk. Indeed, on one hand territorially large but scarcely populated administrative region give the visual impression of a large number of deaths. Whereas on the other hand, very small regions which are densely populated such as big cities are barely visible on maps. Some empirical solutions exist for this latest problem such as displaying cities as larger circles, whose diameter is proportional to population density such as in [47]. However, this method results in partially breaking the geographical continuity and the beauty of evaluating whether a risk crosses multiple territories.

## Statistical analysis of cancer maps

An epidemiological atlas should not simply report the thematic maps but ought to include a number of descriptive and analytical statistics.

**Figure 1.**
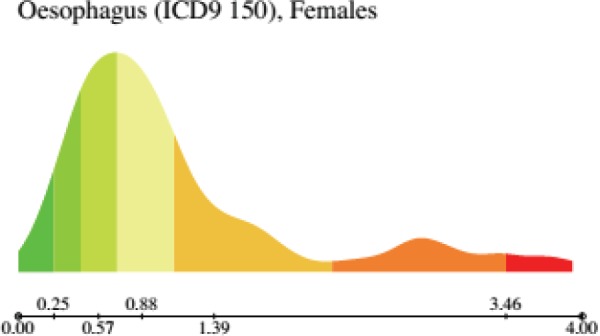
Histogram of the distribution of oesophagus cancer (ICD9 150, corresponding to ICD10 C15) mortality in females in EU and EEA. Note that the distribution is clearly bimodal. Note that, in the cancer-sex relative scale, the colouring map derives from the quantiles of this distribution. *From: P. Boyle and M. Smans (2008). Atlas of Cancer Mortality in the European Union and the European Economic Area 1993–1997. IARC Scientific Publication n°159. Lyon, France: International Agency for Research on Cancer.*

The first basic descriptive statistics are contained in the histogram of the reported values, which is seldom symmetrical and sometimes is either bi- or multi- modal (Figure 1) or may exhibit a long ‘queue’. The latter phenomenon denotes the existence of a number of ‘outlier’ areas where the mortality rate is very large.

To each county one can associate an unknown specific relative risk, which in following [41, 50] has a skewed distribution that can be inferred by Bayesian methods. Particularly important is the estimate of the Standard Deviation of the Relative Risk (RRSD) which provides useful information on the span of the RRs in a map.

In a multination atlas one should also take into account the fact that the counties are naturally grouped by nations, and there can be risk factors that are nation-specific. Thus, it is reasonable to take into account a further level of RR: the one related the RR variability among nations as modelled in [50]. Note however that a nation-associated pattern may be due not to an underlying pattern of causal determinants of cancer, but to the fact that mortality is influenced both by the stage of the disease at diagnosis and by the effectiveness of treatment. Hence the death rate for a cancer of equal incidence (i.e. of diagnosed cases) may be different from one country to another because both treatment and survival rates are nation-dependent [4].

However, although potentially providing interesting information, the distribution histogram and even the RRSD do not take at all into account the spatial structure of the map [50]. Also nation-specific RR model takes only the hierarchy induced by the organisation of the counties in nations into account. On the contrary, the full mutual spatial arrangement of counties is a precious information whose analysis cannot be only limited to the visual inspection of maps.

Spatial statistics is a very active area [49, 22] which has developed a number of tools which have interesting applications in epidemiology [32] (Note that the spatial structure underlying a thematic map is quite elementary because, in practice, it is a network of entities, the counties, related between them by the relation of neighbouring). This is reminiscent, for example, of other kinds of networks such as social networks like Facebook or Twitter (where the neighbours are called respectively, friends and followers). Note that the concept of neighbourhood is epidemiologically relevant since ‘areas which are close to each other in geographical space may share common environmental, social or demographic factors influencing the incidence or outcome of disease’ [31].

Some of the most important spatial statistics applied to thematic maps have the aim of providing a quantitative translation of ‘visual feeling’ one has by visually inspecting a map.

First, by inspecting a map one can spot some clusters of particularly large/small mortality rate. This is very *good* but when dealing with a map with a very large number of areal units, one may not note some of these clusters. To automatically detect them various statistical tests have been developed [17, 32]. In this kind of statistical investigation where the significance of many rates form many small administrative units, to further investigate the issue of multiple comparisons is of great relevance and must be taken into account [28, 2].

**Figure 2.**
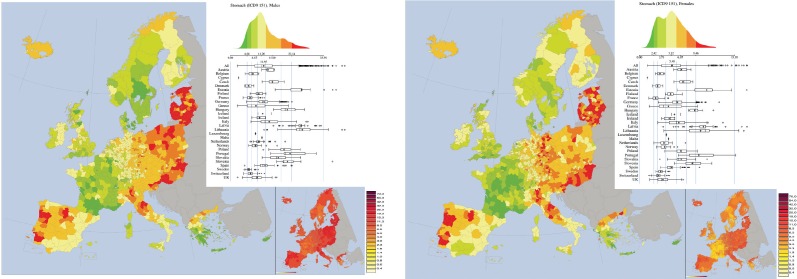
Mortality caused by stomach cancer (ICD9 151, corresponding to ICD10 C16) in EU and EEA in 1993–1997 in males (upper panel) and females (lower panel). Note the large areas of homogeneous colouring, which is mirrored in a large spatial autocorrelation statistics (Moran Index). Note that the two maps appears quite similar, which is mirrored by a large spatial mutual correlation statistics (bimap Moran Index). From: P. Boyle and M. Smans (2008). Atlas of Cancer Mortality in the European Union and the European Economic Area 1993–1997. IARC Scientific Publication n°159. Lyon, France: International Agency for Research on Cancer.

In many cases, observing a thematic map one notes very large areas where the colours of the areal units are similar (Figure 2). Roughly speaking one is led to think that among the values there is a form of ‘correlation’. This led to the formulation of the concept of spatial autocorrelation, which can be defined as follows: ‘the phenomenon where spatial proximity’ of areas ‘is matched by value proximity’ [2]. In order to quantify this spatial autocorrelation has been developed a statistics named Moran’s index I [40, 2]. It linked to a particular type of scatterplot called Moran’s scatterplot [2, 50]. In the Moran’s scatterplot, each geographic unit is represented by a point, whose X coordinate is the value of the mortality in that area and the Y coordinate is equal to the average value of the theme in the bordering areas. The Moran index is simply the correlation coefficient of the scatterplot. If the index I is close to one then the map is very correlated, whereas if it is close to zero in the map there are large areas of random spatial fluctuations. Note that, by definition, in the computation for this statistics one cannot include ‘mono-province’ islands because they have no neighbours.

The Moran scatterplot can also be employed to detect spatial clusters, either by visual inspection or by applying algorithms for data grouping (i.e. clustering of general, non-spatial data), such as the K-means algorithm.

In a cancer atlas it is natural to compare a pair of maps for the mortality of men and women. They may visually appear similar (Figure 2) or dissimilar, but it is necessary to quantify their degree of similarity or dissimilarity. The measure of spatial similarity has been introduced in [50] by defining the two-maps Moran’s scatterplot and the bivariate Moran’s index, which are suitable extensions of the Moran’s scatterplot and of the Moran’s index respectively. The bi-maps Moran’s scatterplot of two maps, for instance let us say A and B is the scatterplot wherein each area *i* is represented by a point whose abscissa is the average value of A mortality measured in the set formed by area *i* and (if they exist) all its neighbouring areas; the ordinate is correspondingly the average value of B mortality measured in the set formed by area *i* and (if they exist) all its neighbouring areas. The Moran’s bivariate index is thus defined as the correlation coefficient associated to the two-maps Moran’s scatterplot. Note that, by definition, in the computation for this statistics one can include ‘mono-province’ islands. If the correlation is close to zero, then there is no common spatial structure for cancer mortality between men and women; if the correlation is close to one, then the two maps have very similar spatial distribution of mortality. Of course, the two-maps Moran scatterplot and the bivariate Moran’s index can be used to compare also two maps referring to two different cancers, for example Breast cancer in women versus ovary cancer in women.

## The geographic impact on cancer mortality of tobacco and alcohol

As we mentioned in the introduction, cancers occur in individual and not in regions and the process from geographic patterns of cancer mortality to possible inferences concerning old and new risk factors is extremely complex, so that the interpretation of atlases must be done very cautiously. On the contrary it is extremely useful to read a cancer atlas in light of the knowledge of general patterns of well-known risk factors for cancer.

Here we focus on the two most important determinants of cancers in humans: tobacco smoking and alcohol consumption.

### Tobacco smocking epidemics: the geographical impact

Tobacco is the causative agent of one third about of all cancers [13]. In particular, it has been estimated that about 80–90% of lung cancers are caused by tobacco [13]. However, tobacco is a strong risk factor for many other cancers: lung, oral cavity, pharynx, larynx, oesophagus, pancreas, stomach, kidney, urinary bladder, cervix uteri, and other cancers. Moreover in general, as confirmed by WHO, tobacco is the most important preventable cause of death in the world [61].

Tobacco smoking mainly affects males, the major tobacco-related mortality is observed in males, although in many countries this males–females disparity is unfortunately decreasing, also because women have been identified by tobacco industry as a vulnerable group.

A striking aspect in tobacco smoking epidemics is that it unequally affects world population following a true ‘negative socioeconomic gradient’–both at aggregate level and at individual level—because its burden is disproportionately heavier in lower social groups [5]. The consequence of this fact and of the abovementioned role of tobacco in the most important preventable causes of death is that the higher is the socioeconomic group the larger is the life expectancy [5]. This scenario is even worsened by the lower degree tobacco stopping motivation and difficulties in accessing to tobacco cessation services, both largely documented in lower socioeconomic groups [5].

As a consequence it is reasonable to expect that at a geographic level these issues are mirrored by disparities across countries and within them.

### The ‘endemic’ alcohol-consumption

It has been estimated that 5.1% and 1.3% of all cancer cases respectively in men and women are caused by alcohol [6]. In particular, there are evidences of a causal dose-dependent relationship existing between alcohol consumption and cancers of oral cavity, pharynx, larynx, oesophagus, liver, colon and rectum, and female breast cancers [7]. In women, breast cancers are 60% of alcohol-related cancers [6]. Both duration of drinking habits and genetic signatures are important [7].

The patterns of alcohol drinking during the week are very linked to national and cultural factors and may substantially vary across nations and often also within a nation [53]. As Zatonski and colleagues stressed [43] that the patterns of the types of alcohols that individuals uses to drink may have an influence on the relative risk for some of the above-mentioned cancers (drinking beer or wine, versus drinking spirits; among spirits: fruit based spirits versus vodka-like spirits etc.).

Finally, alcohol consumption and tobacco smoking can synergise, so that for example the interaction of these two factors is largely statistically significant for the risk of oesophagus, squamous cellular carcinoma in central and east Europe [24] resulting in a strong increase of risk for this cancer. As far as this part of Europe is concerned, a substantial burden of all alcohol-drinking related cancers has been noticed in Central and Eastern Europe [7, 6, 24, 43], where the fraction of cancer cases attributable to alcohol is larger than in the other areas of world [6].

Last but not the least, the drinking habits of women are often and in a nation-dependent manner different from those of men. Indeed on an average women in many European countries drink smaller quantities of alcohol than men [53] and often different kind of beverages [53].

## Maps of cancers related to tobacco-smoking or to alcohol consumption or to both

**Figure 3.**
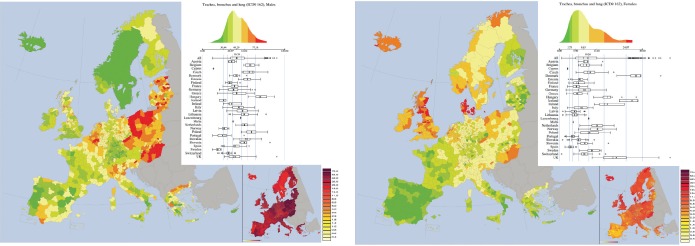
Mortality caused by Trachea, bronchus, and lung cancers (ICD-9 162, corresponding to ICD10 C33 and C34) in EU and EEA in 1993–1997 in males (upper panel) and females (lower panel). See comments in section 4.3. From: P. Boyle and M. Smans (2008). Atlas of Cancer Mortality in the European Union and the European Economic Area 1993–1997. IARC Scientific Publication n°159. Lyon, France: International Agency for Research on Cancer.

*Trachea, bronchus, and lung cancers (ICD-9 162, corresponding to ICD10 C33 and C34)* (Figure 3). This cancer has the largest rates in males and the third-largest in females in the EU–EEA area in the period considered in the atlas [10]. Although mortality rates in males were affected by a large geographical variation, they were constantly larger than the ones observed in females [10].

Among nations the largest mortality rates in males were observed in Hungary (84.8), Poland (71.4), Belgium (69.1); the smaller rate was that of Sweden (22.3), preceded by Portugal (29.3), Iceland (26.2), and Norway (31.5). In females, the largest rates were observed in: Denmark (27.7), Iceland (26.2), and United Kingdom (20.5); the smallest rates were those of Spain (3.9) and Portugal (4.6).

Apart these large variations between countries also within all the countries remarkable changes were observed.

A striking feature of the atlas for this cancer is the cluster of higher rates formed by eastern countries and north-east of Germany. Another cluster is composed by north-west of France, Belgium, Netherland, and some western areas of Germany. A cluster of low rates is formed by Sweden and Norway. In the geographical distribution of mortality for females, Iceland, United Kingdom, Ireland, Denmark, and south of Scandinavia form a cluster of larger mortality, whereas the Iberic peninsula (Spain and Portugal ) forms a cluster of very small mortality rates., which is also present in south of Italy, where indeed the largest regional variability has been observed.

This cancer was characterised by a strong spatial autocorrelation with Moran Index equal to 0.76 for males and 0. 79 for females.

The map for lung cancer clearly follows the *past* patterns of smoking habits between and within European Countries and within each of them. This is true both for regions with large number of smokers, but also for regions and nations where either the prevalence of smokers was traditionally low (e.g. Spain, Portugal, and South Italy for females) but also for regions and nations that were very active in fighting smoking for many decades, such as Scandinavia. Quite interestingly, the two maps of this cancer denote quite different patterns between males and females in some countries such as the UK, Denmark, and Iceland.

**Figure 4.**
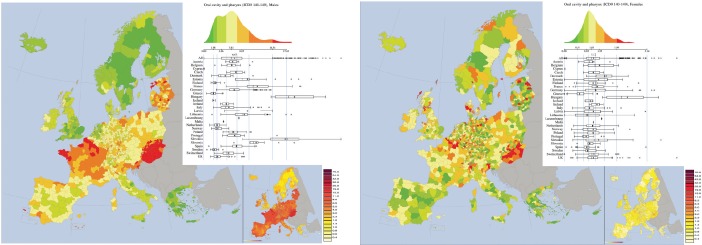
Mortality caused by oral cavity and pharynx cancer (ICD 9 140–149, corresponding to ICD10 codes from C00 to C14) in EU and EEA in males (upper panel) and females (lower panel). See comments in section 4.3. From: P. Boyle and M. Smans (2008). Atlas of Cancer Mortality in the European Union and the European Economic Area 1993–1997. IARC Scientific Publication n°159. Lyon, France: International Agency for Research on Cancer.

*Oral cavity and p harynx cancer (ICD 9 140–149, corresponding to ICD10 codes from C00 to C14)* (Figure 4). The abovementioned factors and patterns are well mirrored in the map of mortality of oral cavity and pharynx, where a cluster corresponding to the higher mortality is constituted by Hungary and Slovakia, and another by Baltic Countries. Large mortalities are also seen in Slovenia and in the North of France. France shows two interesting geographical patterns. The first is a north-south gradient, which probably follows the gradient of drinking habits. Following the terminology adopted by [43] the drinking pattern in the South of France is Mediterranean, whereas the northern one is more Nordic. Moreover, the map well reproduces the drinking differences between the North of France and Belgium. The higher mortality rates of France abruptly end at the border with Belgium (where the risk halves). A gradient is also observable in Italy, from north to south, which is mirrored by the highest RRSD of the map. Scandinavia and Greece form two clusters of lower mortality. Finally, also the UK forms a cluster of lower mortality, whose interpretation is quite difficult. The visual impression of a map with large areas of similar rates is confirmed by the spatial autocorrelation statistics. Indeed, the Moran index of 0.71 confirms a substantial spatial autocorrelation.

Observing the map for females, one might have the false impression of the presence of important patterns. In reality they are mainly caused by the narrow range of the mortality rates which are also low in absolute values. The only real cluster is the one corresponding to Hungary. The fallacy of the visual patterns is confirmed by the fact that the Moran Index is 0.24, one of the smallest of the atlas.

**Figure 5.**
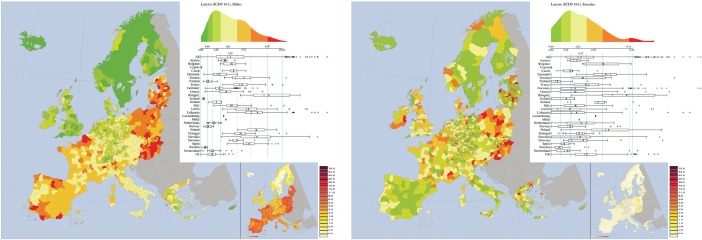
Mortality caused by larynx cancer (ICD 9 140–149, corresponding to ICD10 C32) in EU and EEA in 1993–1997 in males (upper panel) and females (lower panel). See comments in section 4.3. From: P. Boyle and M. Smans (2008). Atlas of Cancer Mortality in the European Union and the European Economic Area 1993–1997. IARC Scientific Publication n°159. Lyon, France: International Agency for Research on Cancer.

*Larynx (ICD-9 161, corresponding to ICD10 C32)* (Figure 5). As for oral cavity and pharynx in males, Hungary and Slovakia exhibit large mortality rates for males. Here also Poland shows similar behaviour. Again, also other Nordic countries as well as UK form two clusters of lower mortality rates which are also common in most of Germany.

Larynx is a very rare cancer for females, so any pattern in the map is mostly an artefact because of the low numbers. Similarly to oral cavity and pharynx, the map for larynx cancer for males has a large spatial autocorrelation (Moran index = 0.71), whereas no autocorrelation is found for females (Moran index = 0.21). No association is found between the male and female maps (bivariate Moran index is 0.29).

**Figure 6.**
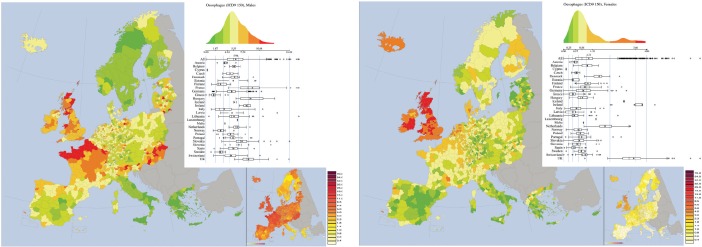
Mortality caused by oesophagus cancer (ICD-9 150, corresponding to ICD10 C15) in EU and EEA in 1993–1997 in males (upper panel) and females (lower panel). See comments in section 4.3. From: P. Boyle and M. Smans (2008). Atlas of Cancer Mortality in the European Union and the European Economic Area 1993–1997. IARC Scientific Publication n°159. Lyon, France: International Agency for Research on Cancer.

*Oesophagus cancer (ICD-9 150, corresponding to ICD10 C15* (Figure 6). For oesophagus cancer in males, Northern France, Scotland, and Hungary are three clusters of higher mortality rates; the centre-south of Italy, Greece and Scandinavia instead form three clusters of lower mortalities. France is also characterised by a marked north-south gradient.

For females, the UK and Ireland form a cluster of larger values for mortality rate clearly visible in the histogram of mortalitiesis bimodal. The Iberic peninsula (Spain and Portugal) forms a cluster of lower values.

This site was the one showing the highest spatial autocorrelation for female (Moran Index = 0.82), and it is quite large also for male (Moran Index = 0.70). Moderate mutual spatial correlation between the maps for the two sexes (0.56).

**Figure 7.**
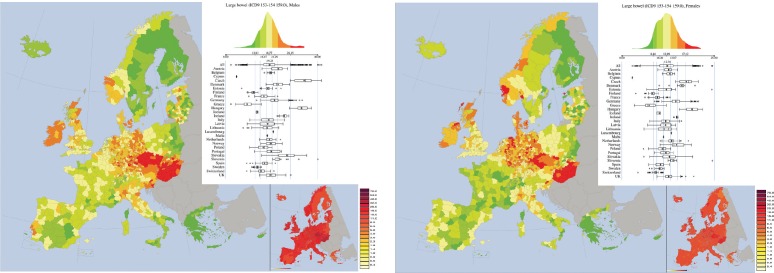
Mortality caused by Large bowel Cancers (ICD-9 153, 154 and 159.0, corresponding to ICD10 C18) in the EU and EEA in 1993–1997 in males (upper panel) and females (lower panel). See comments in section 4.3. From: P. Boyle and M. Smans (2008). Atlas of Cancer Mortality in the European Union and the European Economic Area 1993–1997. IARC Scientific Publication n°159. Lyon, France: International Agency for Research on Cancer.

*Large bowel cancers (ICD-9 153, 154 and 159.0, corresponding to ICD10 C18)* (Figure 7). As far as males are concerned, Czech and Hungary form two important clusters. Clusters of lower mortality rates are observed in Greece, South Italy, Finland, and Sweden.

For females, similar patterns are observed, but here also France and Switzerland form another cluster of smaller mortality rates.

In both maps: among the smaller regional variation (RRSD is 0.25 for males and 0.24 for females) and among the larger spatial autocorrelation (0.74 for males and 0,7 for females). There is very large mutual correlation between the male and female maps, with bivariate Moran Index equal to 0.83.

**Figure 8.**
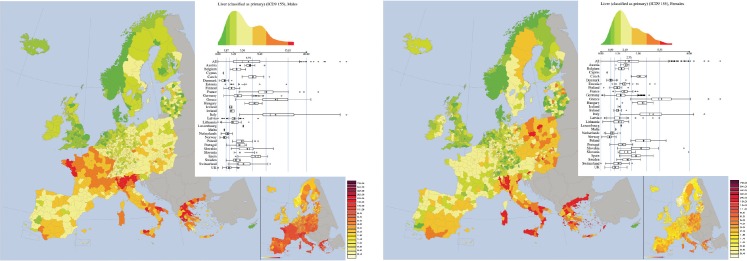
Mortality caused by Liver Cancer (ICD-9 155, approximately corresponding to ICD10 C22) in EU and EEA in 1993–1997 in males (upper panel) and females (lower panel). See comments in section 4.3. From: P. Boyle and M. Smans (2008). Atlas of Cancer Mortality in the European Union and the European Economic Area 1993–1997. IARC Scientific Publication n°159. Lyon, France: International Agency for Research on Cancer.

*Liver Cancer (ICD-9 155, approximately corresponding to ICD10 C22)* (Figure 8). In the map referring to males, Greece and France + Italy form two clusters of large mortality rates, whereas clusters of mortality rates are formed by UK and by Sweden + Norway.

In the map for females, large rates are observed in Italy and Greece, but not in France. It is worth noting that large mortality rates characterise the whole national border of Italy, depicting a cluster identical to the nation. Large spatial autocorrelation is obtained by the Moran Index, which is 0.80 for males and 0.74 for females. Moreover, the visual similarity of the maps for males and females is confirmed by the bivariate Moran index, which is equal to 0.73.

We reiterate that for this cancer not only alcohol drinking is a risk factor, but also chronic hepatitis B and C, which are endemic in some part of Europe (e.g. some regions of South Italy).

## The case of mapping breast cancer mortality in women

**Figure 9.**
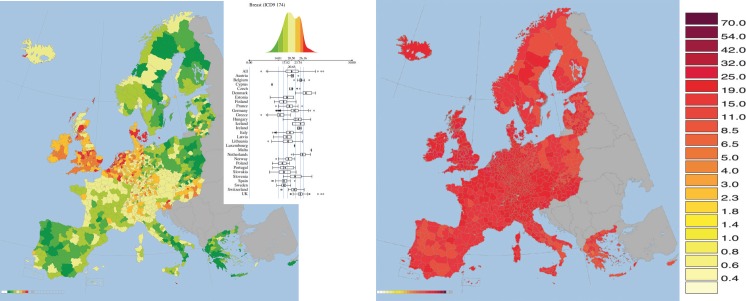
Mortality caused by Breast Cancer (ICD-9 155, corresponding to ICD10 C50) in 1993–1997 in EU and EEA. Upper panel: map plot in the cancer-sex relative scale; lower panel: map plot in the absolute scale. See comments in section 5. From: P. Boyle and M. Smans (2008). Atlas of Cancer Mortality in the European Union and the European Economic Area 1993–1997. IARC Scientific Publication n°159. Lyon, France: International Agency for Research on Cancer.

Breast cancer in the studied period (1993–1997) was the most common form of cancer for women and no countries showed a low mortality rate (Figure 9). The largest rate (27.4) was observed in Denmark, whereas the lowest was observed in Greece (14.8). The rates between countries had some variability, whereas internally to the countries they tended to be quite uniform. The map for this cancer site was highly autocorrelated showing the second largest Moran index of the atlas (0.70).

Moreover, the map of breast cancer has some similarity with that of ovarian cancer, which is confirmed by a bivariate Moran index (spatial correlation between the two maps) of 0.48. For example both maps reported: lower than average rates in Greece, Italy, South of France, Spain, and Portugal; higher than average rates in Denmark, Netherlands, Belgium, United Kingdom, and Ireland.

No known aetiological factors of breast cancer in women can easily explain the map of this cancer.

**Figure 10.**
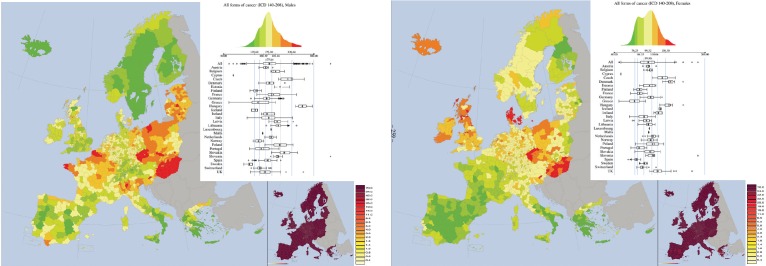
Mortality caused by all forms of Cancersin EU and EEA in 1993–1997 in males (upper panel) and females (lower panel). See comments in section 6. From: P. Boyle and M. Smans (2008). Atlas of Cancer Mortality in the European Union and the European Economic Area 1993–1997. IARC Scientific Publication n°159. Lyon, France: International Agency for Research on Cancer.

However, it is only when looking at the absolute map of breast cancer mortality (Figure 10), i.e. the one adopting the same scale for all kinds of cancers, that one gets the real big picture. It is noted that the breast cancer in women is one of the most uniformly spatially distributed cancers. This denotes a tendency of uniformly large levels of breast cancer mortality for women. This is concordant with the observed convergence of mortality rates in EU, reported in [65].

Breast cancer in women is a good example for stressing the importance of reporting both a map in relative scale and a map in absolute scale.

## All forms of cancers

The mortality rates for all forms of cancer were higher in males than in females in all countries of EU–EEA. Highest mortalities in males were observed in Hungary (268), Czech Republic (228), Slovakia (218), and more in general in the largest part of former communist countries. In western Europe the largest rates were observed in Belgium (194) and in France (188). The smallest rates were in Sweden (121), Iceland (138), and in Finland (139).

In females, the highest rates were observed in Denmark (139), Hungary (138), and in Czech Republic (125). The lowest rates were localised in Greece (76), Spain (78) and Portugal (84).

Eastern countries, linked though Slovakia to Northern Italy, formed a cluster of large mortality rates for males. Another cluster includes the North of France and Belgium. The region of Bretagne (France), forms another cluster. Clusters of low mortality rates are in Scandinavia and Finland, and in southern Europe (Portugal, most of Spain, the South of Italy, Greece).

For females, Hungary forms a distinct cluster of large values attached to Czech Republic and West of Poland. Another cluster is formed by Denmark. Large values are also observed in the UK and Ireland. The southern cluster of low mortality rates observed for males is also observed for females, but it also extends to most of France.

Large within countries variability are observed for males, for example in Italy and France.

Not surprisingly, in males the patterns observed for all kinds of cancers partially follow those of tobacco- and drinking-related cancers, whereas for females one notices the influence of tobacco-related cancers and of breast cancer.

## Concluding remarks

In this review we illustrated the basic concepts of cancer mortality mapping, as well as the motivation underlying the production of a Cancer Mortality Atlas.

A key concept we wanted to transfer to the reader is that the ‘per se’ interesting spatial pattern that can be evidenced by a Cancer Mortality Atlas (but the concept remains valid for a general Atlas of a Disease) is only the first step of a long process of identifying the cancer causes underlying the evidenced pattern of increased mortality, or conversely the good behaviours that led to a spatial pattern of low mortality. This is a long and uncertain process where success is not guaranteed.

However, we must also specify that this is not a specific issue of cancer mapping, but a general problem in cancer epidemiology. As stressed by Doll and Peto (1981) from whatever kind of data the identification of cancer causes is complex, long, and quite often the first hypothesised putative cancer causes are not the correct ones [63].

Another concept we stressed is that a cancer atlas is not only a visual tool, an atlas containing only maps would be of limited utility. Indeed, the visual intuition coming from the inspection of maps must be complemented and validated by rigorous statistical analyses. The underlying statistical methodologies are fascinating, complex, and to some extent *in fieri*. Thus a detailed description of them would go well beyond the aims of this review. The interested reader is invited to read the references of section 3.

In this work, we mainly made reference to the last Cancer Mortality Atlas of EU and of the European Economic Area [10]. Since that Atlas is quite large, we decided to provide some examples by following two guidelines. First, we illustrated the maps of some of the cancers that are more importantly caused by two well-known behavioural cancer causes, i.e. smoking tobacco and drinking alcohol. Then we went onto illustrate the case of breast cancer in women, both because it is the most important cancer affecting women and because it illustrates the importance of including in a mortality atlas also maps coloured in an absolute scale. Finally, of course we illustrated the case of the map for all types of cancers.

Finally, it is worth reiterating that usually a cancer atlas illustrates data that are temporally distant from the year of publication of the atlas [47], and this is for two reasons. The first is that the data collection and validation is a complex process that often requires years; the second is that producing an atlas is nontrivial and it involves many delicate steps like defining the geographical units, choosing the statistical methodologies, getting the data from national health authorities, handling them, and then validating, elaborating and visualising these data. A reduction of the delay from the data and the publishing of an atlas might be feasible thanks to the recent progresses epidemiology, computer graphics, and of spatial statistics.

The last Cancer Mortality Atlas of Europe has been published eight years ago and refers to a remote period of time (1993–1997), and we hope that soon the production of a new Atlas, referring to a more recent period will be published. Apart from its intrinsic relevance, such a new atlas would allow statistical comparisons with the data shown in the previous atlases, in order to verify whether and if how much spatial patterns changeover has occured with time. This would potentially trigger new and perhaps interesting epidemiological questions.

## Dedication

This paper is dedicated to the memory of our beloved friend, IEO colleague and co-author Chiara Mazzetta (1973–2010).
